# Lithium Thiostannate Spinel Thin Films as Potential Photovoltaic Absorbers

**DOI:** 10.1002/nap2.70330

**Published:** 2026-10-23

**Authors:** Naini Jain, Kristina Vlášková, Lucie Landová, Neda Neykova, Dmytro Vorontsov, Oleksandr Romanyuk, Lukáš Horák, Milan Dopita, David Bušek, Amalraj Peter Amalathas, Jakub Holovský

**Affiliations:** ^1^ SOL‐MAT Lab Faculty of Electrical Engineering Czech Technical University in Prague Prague Czech Republic; ^2^ Institute of Physics Czech Academy of Sciences Prague Czech Republic; ^3^ Faculty of Mathematics and Physics Charles University Prague Czech Republic; ^4^ Department of Physics Faculty of Science University of Jaffna Jaffna Sri Lanka

**Keywords:** Li_1.66_CuSn_3.33_S_8_films, phase‐pure, photo‐absorber, photoconductivity

## Abstract

Quaternary lithium thiostannate spinels represent a promising class of non‐toxic earth‐abundant semiconductor absorbers for photovoltaic applications, reported to be air‐stable in bulk form. To the best of our knowledge, no attempts to prepare their thin films and evaluate their photoresponse have been reported. Here, we demonstrate the first successful deposition of nanocrystalline Li_1.66_CuSn_3.33_S_8_ spinel films (∼350 nm thickness) via pulsed laser deposition (PLD). We observe that the crystal structure of the PLD target governs the structure and optoelectronic properties of the resulting film. A phase‐pure spinel target yields a homogeneous layer with a high degree of crystallinity, strong optical absorptance with a clear absorption edge at ∼1.5 eV and low sub‐bandgap values, as determined by photothermal deflection spectroscopy. The photoluminescence peak centred at 836 nm, consistent with the absorption band edge, together with measurable photoconductivity, confirms semiconducting character. Therefore, we identify the PLD‐deposited Li_1.66_CuSn_3.33_S_8_ films as a candidate for a photo‐absorber material, while noting that further improvements in film crystallinity, sulphur stoichiometry and Urbach energy are required before photovoltaic device integration can be assessed.

## Graphical abstract

The first successful deposition of nanocrystalline Lithium thiostannate thin film using the pulsed laser deposition technique, with the first reported photoconductivity and photoluminescence, confirms a non‐negligible carrier lifetime and mobility.

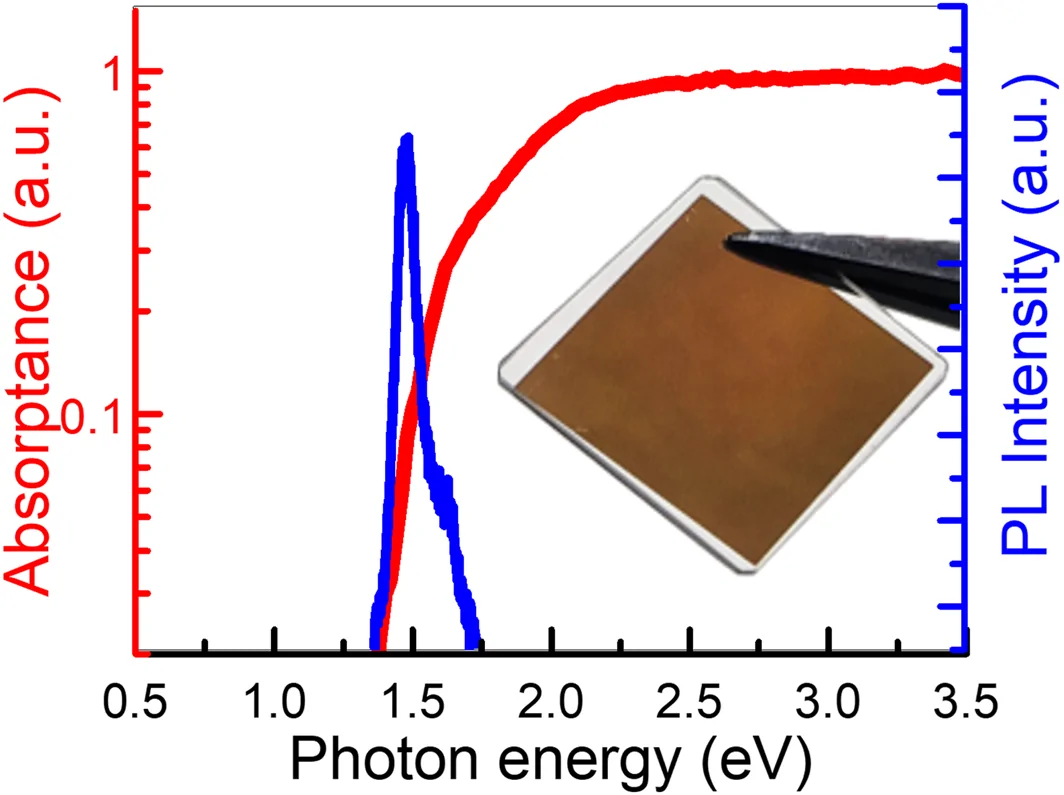

## 1 Introduction

Photovoltaic (PV) technologies are synonymous with the transition toward sustainable energy. Their broader deployment, however, will depend not only on lowering cost but also on improving power output under realistic operating conditions, extending device lifetime and reducing environmental burden. Tandem solar‐cell architectures offer a promising route to higher efficiencies, with crystalline silicon remaining the natural bottom‐cell platform. In this context, the search for a stable, non‐toxic and scalable top‐cell absorber remains an important open challenge.

Organic–inorganic hybrid perovskites (OIHP) have gained significant attention as a top‐cell candidate, owing to their high power conversion efficiencies (26.8%) [1]. The solution‐grown layers exhibit strong absorption, a tunable bandgap and defect tolerance. The latter might be attributed to octahedrally coordinated ionic bonding. Nevertheless, their advantages are hindered by instability under operating conditions [2] and ecological concerns stemming from the presence of lead [3]. This has stimulated research into lead‐free alternatives, including lead‐free halide perovskites [4] or double perovskites Cs_2_AgBiBr_6_ [5] and also monocrystalline materials from the III–V family [6]. Polycrystalline kesterite compounds [7] (CZTS, CFTS etc.), chalcogenide perovskites (BaZrS_3_, SrZrS_3_ etc.) and binary compounds such as sulphides such as SnS [8], Bi_2_S_3_ [9], CuS [10], and FeS_2_ [11] etc. [12]. Despite considerable research effort, these materials remain limited in their success as thin‐film photovoltaic absorbers. The dominant barrier is that these materials rely primarily on tetrahedral covalent bonding, which is more sensitive to crystalline disorder and phase impurities, making phase‐pure thin‐film growth difficult.

Chalcogenide spinels, particularly the quaternary thiospinel A_2_BSn_3_S_8_ family (A–Li, Cu, Ag, B–Fe, Zn, Ni, Co and Mn), have emerged much more recently [13, 14, 15]. The A_2_BSn_3_S_8_ structure combines metal–sulphur tetrahedra (chemical stability) and octahedra (defect tolerance), serving as a great alternative for OIHPs, preserving the tunability of optoelectronic properties, enhancing chemical stability, reducing material toxicity and providing intrinsic defect tolerance [13, 14, 16]. High defect tolerance, which is particularly important for thin‐film technologies, has been predicted by DFT for the related spinel, HgX_2_S_4_ [17]. Lithium (Li), an alkali metal with a small ionic radius of 0.76 Å, is well‐coordinated within the Sn‐S cubic framework. This improves the charge‐carrier mobility, air/moisture stability and maintains charge balance [18]. Quintero et al. [14] have investigated the bulk Li‐thiospinels Li_2_BSn_3_S_8_ (B–Mn, Cu, Fe, Mn, Ni and Co) synthesized using high‐temperature and mechanochemical methods. The copper analogue (Li_1.66_CuSn_3.33_S_8_) would be a promising photovoltaic absorber, owing to its suitable bandgap (1.51 eV) and its low room‐temperature ion conductivity, which limits the mobile ions drift under applied bias and their accumulation at interfaces, which in halide perovskite causes field screening, hysteresis and compositional change [19, 20].

Despite this favourable outlook, thin films of Li_1.66_CuSn_3.33_S_8_ have not yet been reported, and their photoresponse has not been evaluated. Establishing a thin‐film deposition route is therefore a necessary step towards assessing whether these materials can function as photovoltaic absorbers. Pulsed laser deposition (PLD) is a well‐suited technique, as it is a highly non‐equilibrium ablation process ensuring near‐stoichiometric transfer, even when the constituent elements differ substantially in volatility and bonding characteristics [21, 22]. At the same time, for complex sulphides, the structural and phase quality of the ablation target is expected to be critical for obtaining phase‐pure films. Here, we report the first successful deposition of a Lithium thiostannate thin film by PLD, complemented with crystallinity, morphology and optoelectronic analysis.

## 2 Results and Discussions

### 2.1 Morphology and Phase Identification

To achieve phase‐pure Li_1.66_CuSn_3.33_S_8_ thin films, phase‐pure spinel powders, prepared by mechanochemical synthesis, must first be prepared and pressed into the form of the PLD ablation target, schematically demonstrated in Figure 1. Impure targets invariably produce multiphase films; therefore, target preparation was treated as the critical first step. Two target types were prepared by high‐energy ball milling and cold‐pressing of stoichiometric precursor powders (full details in Experimental Methods): a homogenised target (3 h milling) and a phase‐pure target (20 h milling). The systematic structural evolution during homogenization and mechanochemical synthesis was analysed using X‐ray diffraction (XRD) and Raman spectroscopy (see Supporting Information S1: Figure S1). The mechanosynthesised phase‐pure ball‐milled powder exhibits diffraction peak patterns corresponding unambiguously to the *Fd* $\bar{3}$ *m* spinel structure (see Supporting Information S1: Figure S1a). Having established the crystallographic and phase integrity of the target pellets, we loaded the pellet into the portable chamber, avoiding air exposure, and we proceeded with the PLD deposition on a quartz substrate and post‐deposition annealing. The scanning electron microscopy (SEM) images in Figure 2 of the resulting film (∼350 nm thickness) grown from a phase‐pure target reveal a pinhole‐free, fine‐grained, dense nanocrystalline morphology.

**FIGURE 1:**
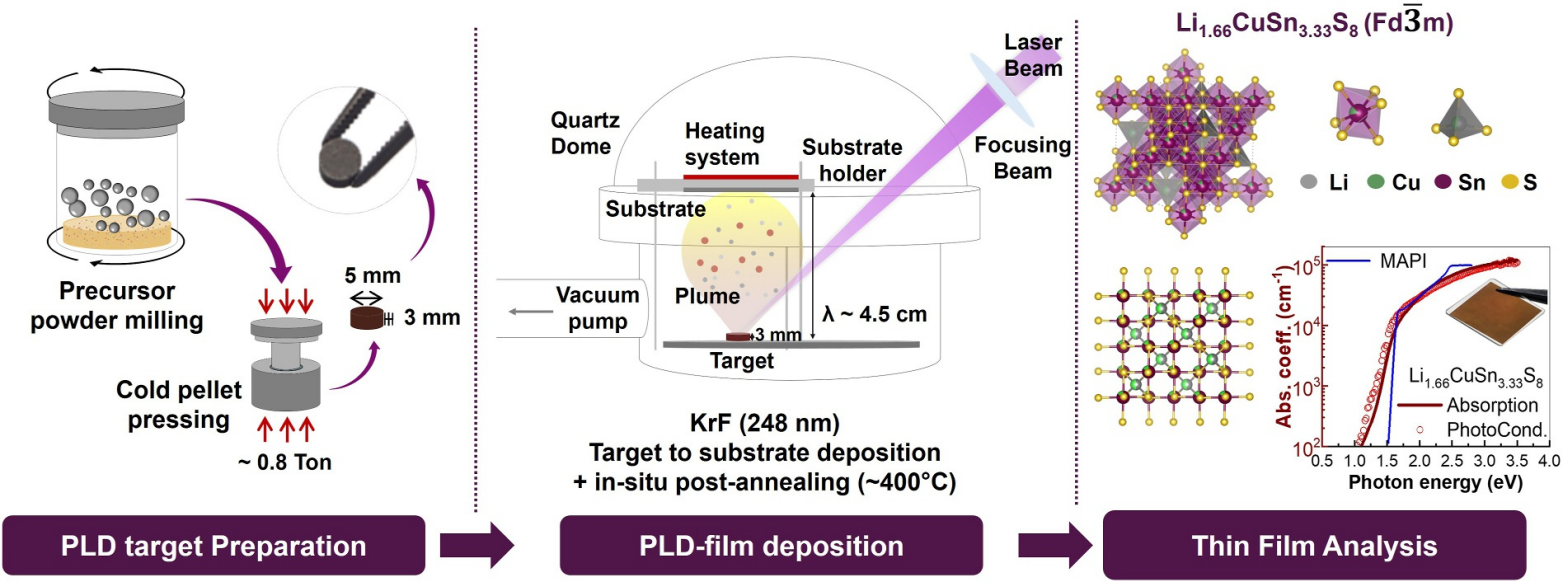
Schematic explanation of the experimental procedure for depositing phase‐pure Li_1.66_CuSn_3.33_S_8_ film.

**FIGURE 2:**
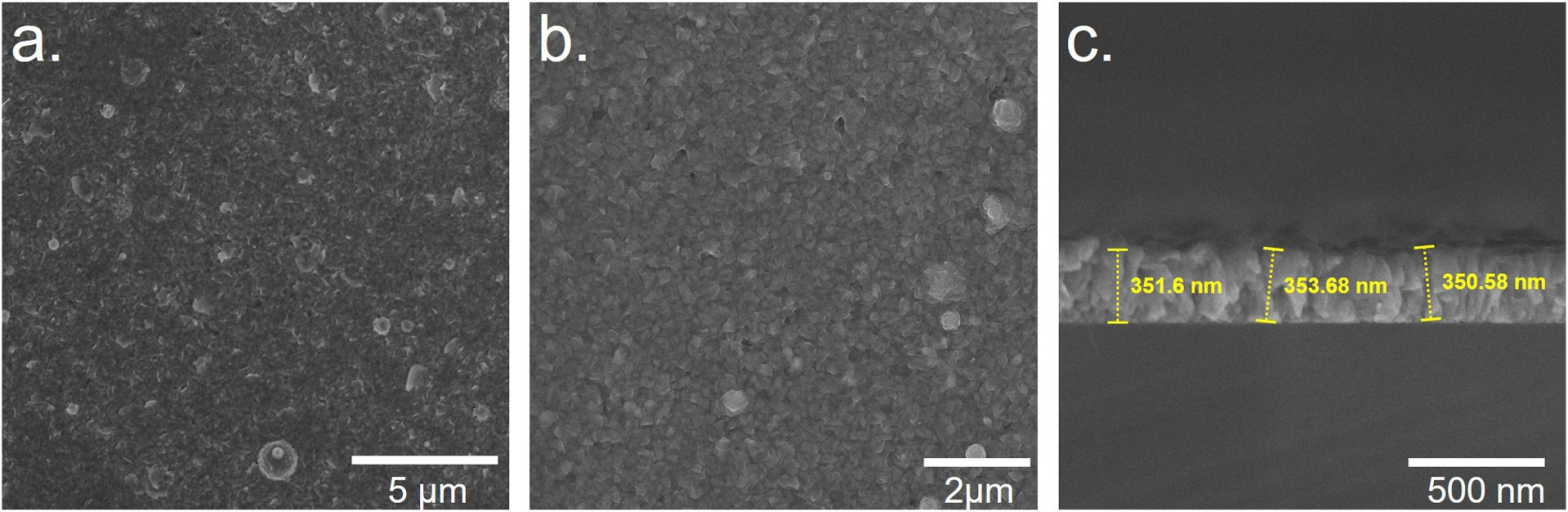
(a–b) Top‐view SEM images on different scales: (a) 5 μm, and (b) 2 μm; (c) cross‐section of the Li_1.66_CuSn_3.33_S_8_ film, schematically showing the nanocrystalline nature with estimated thickness ∼350 nm.

We investigate how the target phase purity governs the crystal structure of the post‐annealed PLD thin films (400°C, 60 min), using XRD. The diffraction patterns originating from both target types are presented in Figure 3 alongside the calculated reference for the cubic spinel space group *Fd* $\bar{3}$ *m*. For the PLD film deposited from only the homogenised pellet, the observed XRD pattern reveals the presence of multiphase reflections, showing the preferential secondary phases (Cu–S, Sn–S etc.). Conversely, for the film with a phase‐pure pellet, the observed diffraction peak patterns confirm the *Fd* $\bar{3}$ *m* spinel structure. The peak positions at (111), (311), (222), (400), (511), (440) and (531) reflections spanning the 2θ range of 10°–65° align closely with the *Fd* $\bar{3}$ *m* reference tick marks, confirming that the PLD from the mechanosynthesised phase‐pure target delivers phase‐pure Li_1.66_CuSn_3.33_S_8_ films without preferential chalcogenide loss or cation segregation. A prominent feature of both diffractograms is the strong preferential enhancement of the (111) reflection intensity relative to its powder reference, a signature of preferential orientation driven by the low surface energy of the (111) spinel planes. Rietveld refinement of the *Fd* $\bar{3}$ *m* structural model, with Sn^4+^ occupying the octahedral 16d site, S^2−^ on 32e and a mixed Cu^+^/Li^+^ distribution across the tetrahedra 8a and octahedra 16d Wyckoff positions, yields cubic lattice parameters of *a* = 10.4(3) Å, in agreement with the reported bulk value for this quaternary spinel. This confirms that the films are stoichiometrically faithful to the target composition.

**FIGURE 3:**
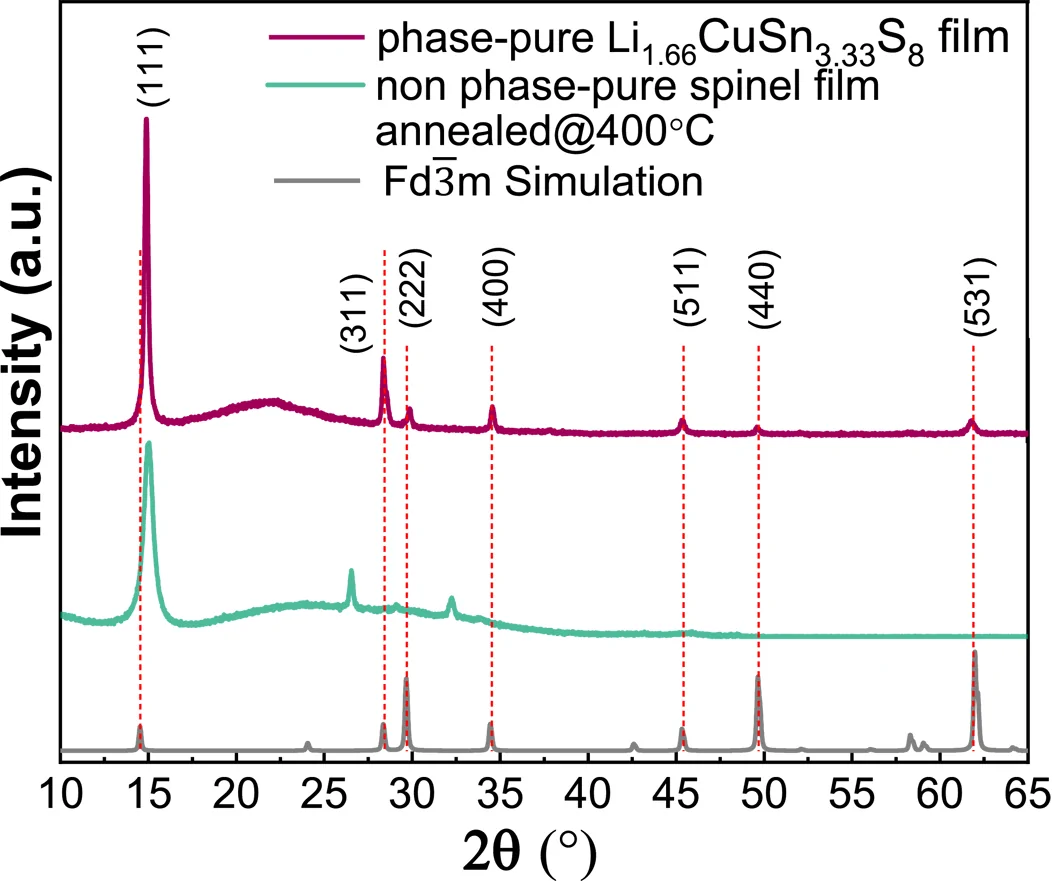
XRD diffraction patterns of the non‐phase‐pure and phase‐pure Li_1.66_CuSn_3.33_S_8_ spinel films, post‐annealed at 400°C.

Whereas XRD integrates the signal from a large area, Raman spectroscopy mapping provides complementary spatially resolved information on phase integrity and crystallinity. Li_1.66_CuSn_3.33_S_8_ spinel belongs to the cubic O_h_7 (*Fd* $\bar{3}$ *m*) space group, with two formula units per primitive cell [23]. Group‐theory analysis at the *Γ* point yields a total of five orders of the Raman‐active modes at the centre of the Brillouin zone [24]: *Γ*_Raman_ *= A*_*1g*_ *+ E*_*g*_ *+ 3T*_*2g*_. The Raman spectra of phase‐pure Li_1.66_CuSn_3.33_S_8_ spinel films is shown in Figure 4a. The spectra were fitted with Gaussian line shapes (*R* ^2^ = 0.98), yielding five resolved peaks, that is, 90 cm^−1^, 180 cm^−1^, 318 cm^−1^, 344 cm^−1^ and 367 cm^−1^. The Raman maps acquired at the two dominant mode frequencies (90 cm^−1^ and 318 cm^−1^) (See Figure 4c‐d) reveal grain‐to‐grain variability in Raman intensity, suggesting microscopic structural heterogeneity. Notably, according to Raman spectroscopy, the Li_1.66_CuSn_3.33_S_8_ spinel films are stable in ambient air (see Figure 4b). To briefly test also the material tolerance to humidity, layers were immersed in deionized (DI) water, HCl (0.1 M), and KOH (0.1 M) solution for 1, 24 and 48 hours and the changes in crystallinity, phase composition and possible oxidation were tracked by Raman spectra (see Figure 4b). For the visual appearance and more details about the stability test, refer to Supporting Information S1: Figures S7 and S8a–c. We could observe that the material remain visually largely intact in DI water and dilute acid, whereas the only changes in Raman spectra were in the reduced intensity of individual modes. In dilute alkaline solution, by contrast, the material degrades drastically.

**FIGURE 4:**
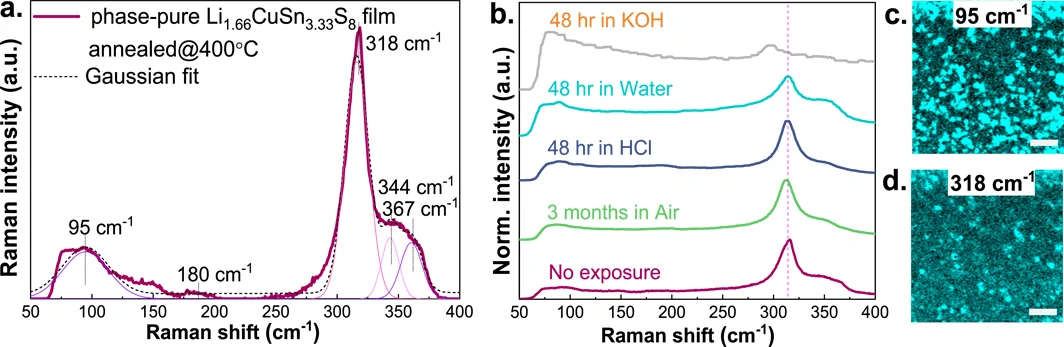
(a) Raman Spectra of pure‐phase spinel films at different Raman modes; (b) Raman spectra of a pure‐phase spinel film, analysing its stability after prolonged exposure to air, water, HCl, and KOH solutions; and (c–d) Raman maps acquired from the respective Raman spectra at the two dominant mode frequencies (90 cm^−1^ and 318 cm^−1^) (Scale bar: 3 μm).

### 2.2 Compositional Analysis

To further assess the stoichiometric transfer from the target pellet to the substrate, the elemental composition of both the phase‐pure spinel pellet (target) and film were investigated by energy‐dispersive X‐ray spectroscopy (EDX) and X‐ray photoelectron spectroscopy (XPS). Figure 5a presents the survey spectra, while Figure 5b shows the high‐resolution Li 1s spectra acquired from both specimens (other elements' higher‐resolution spectra are shown in Supporting Information S1: Figure S2). A remarkable agreement between the spectral features of the target and the deposited film is observed across all measured energy regions, providing direct evidence of successful PLD, in which the chemical composition of the target is faithfully transferred to the film.

**FIGURE 5:**
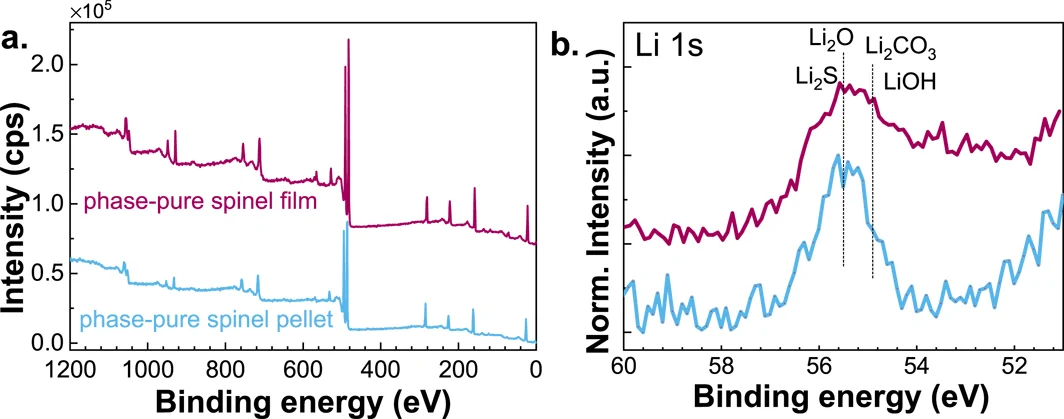
(a) Survey XPS spectra; (b) high‐resolution spectra of Li 1s of the pressed pellet (blue) and the deposited film (deep magenta), indicating a successful deposition process.

XPS analysis confirms the presence of Li, Cu, Sn and S, together with C and O species, in both the film and the target pellet. The broadening of the Li 1s peak is likely due to contributions from multiple Li‐containing surface phases, including Li_2_S, Li_2_O, Li_2_CO_3_ and LiOH, with Li assumed in the Li^+^ sulphide state. Minor oxide species, including Cu–O and Sn–O bonds, may also be present at the surface. In contrast, no sulphur oxide species were detected within the experimental resolution, indicating that sulphur also remains in the sulphide state. Table 1 summarises the atomic concentrations of elements Li, Cu, Sn and S, measured using EDX and XPS (C and O were not involved in the stoichiometry analysis). Both measurements have shown moderate agreement with the required stoichiometric ratio. The deviation mainly in sulphur content is attributed to the material's intrinsic tendency of PLD layers toward sulphur deficiency [25]. This is reflected in the excess of all other elements. Moreover, the XPS results are affected by low sensitivity to Li (see Figure 5b) as well as by various surface effects related to ageing, for example, trapping of Li at the surface in the O^−2^, OH^−1^ and CO_3_^−2^ complexes.

**TABLE 1:** The atomic concentration of the Li_1.66_CuSn_3.33_S_8_ film obtained from EDX and XPS measurements, compared with the required stoichiometric ratio.

Measurement techniques/Atomic concentration (%)	Li	Cu	Sn	S
EDX	N/A	11.6 ± 0.7	26 ± 1	50 ± 0.7
XPS	19 ± 4	6 ± 2	27 ± 3	48 ± 4
Stoichiometric ratio	12	7	24	57

### 2.3 Optoelectronic Properties

The optical absorptance of ∼350 nm thick films was measured by the photothermal deflection spectroscopy (PDS) method (Figure 6). The optical absorption coefficient as a function of photon energy for non‐phase‐pure and phase‐pure Li_1.66_CuSn_3.33_S_8_ spinel films was then evaluated by the Ritter–Weiser [26] equation; see Figure 6a. When going from non‐phase‐pure to phase‐pure material, the absorptance increase above the bandgap and reduction below the bandgap are observed, as well as a shift in the near‐direct optical bandgap from 1.5 to 1.6 eV, see Supporting Information S1: Figure S3a. Especially for the phase‐pure Li_1.66_CuSn_3.33_S_8_ spinel film post‐annealed at 400°C for 1 hour, the absorption edge spans almost three orders of magnitude in the sub‐bandgap regime, revealing a decently sharp band edge. Above the band edge, the absorption coefficient reaches values of ∼10^5^ cm^−1^, fully consistent with those of direct‐bandgap semiconductors. A near‐direct optical bandgap was estimated to be around 1.5 eV using the Tauc plot analysis (with exponent *n* = 1/2, corresponding to a direct allowed transition) for phase‐pure Li_1.66_CuSn_3.33_S_8_ spinel films (See Supporting Information S1: Figure S3a), in close agreement with the value of 1.5 eV reported by Quintero et al. for phase‐pure bulk crystals [14].

When comparing the measured bandgap of the material with classical semiconductors, it will exhibit photoluminescence (PL) from the bandgap edge, and the PL intensity will be a measure of excess carrier lifetime. PL measurements of a freshly prepared phase‐pure Li_1.66_CuSn_3.33_S_8_ spinel film reveal the PL emission signal centred at approximately 836 nm (∼1.5 eV), see Figure 6b (amber curve). This emission energy is in close agreement with the direct optical bandgap of ∼1.5 eV extracted from Tauc plot analysis of PDS data; see Supporting Information S1: Figure S3a. The spectral line shape and photon energy can be more accurately attributed to band‐to‐band radiative recombination by application of the optoelectronic reciprocity theorem [28, 29] (see Supporting Information S1: Equation 1) relating PL to absorptance, see Supporting Information S1: Figure S4. The observation of measurable PL is evidence of semiconducting properties of the material and a relatively low recombination rate. However, when we tracked the time evolution, 15 days later, the PL signal was damped. The degradation of this emission would likely be because of partial oxidation or structural rearrangement at grain boundaries. This observation underscores the need for passivation or capping strategies in future device architectures. The film deposited from the homogenised pellet exhibits a markedly blue‐shifted PL emission centred at 748 nm (∼1.6 eV), see Supporting Information S1: Figure S5, corresponding to its observed bandgap shift.

**FIGURE 6:**
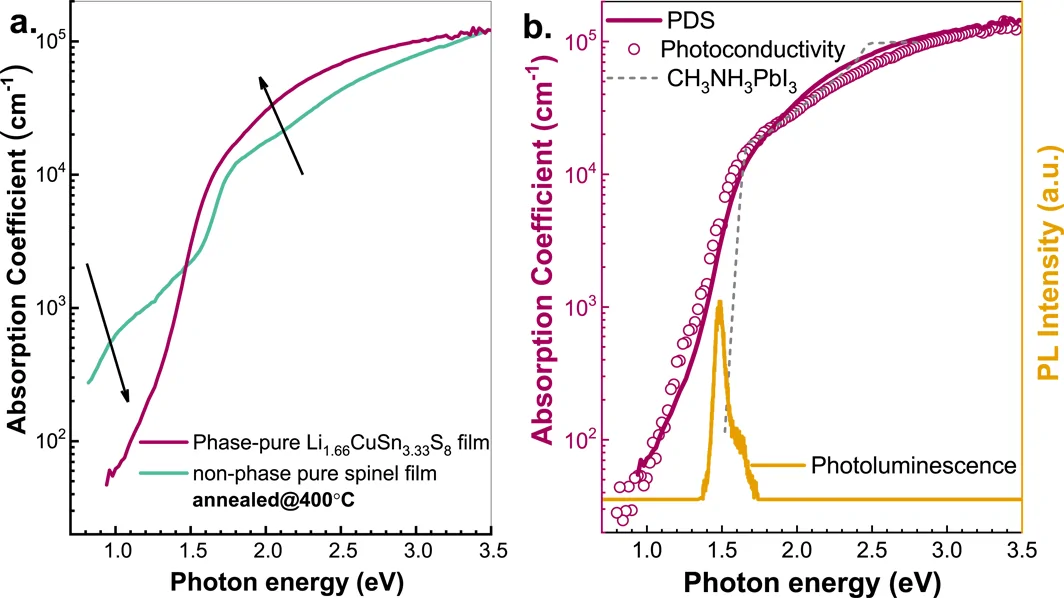
(a) The optical absorption coefficient (α) as a function of photon energy for non‐phase‐pure and phase‐pure Li_1.66_CuSn_3.33_S_8_ spinel films. (b) The PDS and photoconductivity were recalculated into the optical absorption coefficient together with photoluminescence spectra, for phase‐pure Li_1.66_CuSn_3.33_S_8_ spinel films. For comparison, the absorption coefficient of MAPI obtained from PDS after surface scattering correction [27] is given.

Another important property of solar cell absorbers, apart from a low recombination rate, is the carrier mobility. The product of carrier mobility and carrier lifetime is a measure of photoconductivity. Photoconductivity was measured using evaporated coplanar gold contacts with 0.5 mm spacing, under a 20 V bias, with the monochromatic illumination from the same setup as for PDS was used, complemented by the Keithley 428 current preamplifier (amplification of 10^9^ V/A). Measurable photoconductivity therefore also confirms a non‐negligible carrier mobility, see Figure 6b (deep magenta circles). The photocurrent onset closely tracks the PDS absorption edge above the band gap. Whereas the PDS is measuring the total absorptance, photoconductivity registers only the absorptance contributing to the electrical signal, that is, the absorptance attributed to the photoconductivity, explaining the small discrepancies between the PDS absorptance and the photoconductivity signal.

The sub‐bandgap absorption is analysed within the Urbach formalism [30], in which the absorption coefficient (*α*) below the absorption edge follows an exponential dependence on photon energy of the form *α = α*_*0*_ exp[*(hν − E*_*g*_ *)/E*_*u*_], where *E*_*u*_ is the Urbach energy. When going from homogenised to the phase‐pure spinel, the absorption onset progressively sharpens, and *E*_*u*_ decreases from ∼120 to ∼82 meV, as shown in Supporting Information S1: Figure S3b. This reduction in Urbach energy reflects a decrease in the density of sub‐bandgap tail states and is consistent with improved long‐range structural order in the absorber layer. Nevertheless, E_u_∼82 meV remains significantly higher than the values reported for high‐efficiency photo‐absorber materials such as silicon, III–V or MAPI, with values below 15 meV [31]. If there are no other spatially localised defects, the open‐circuit voltage (V_OC_) of a solar cell attainable by the theory, using band filling correction [32] can be ∼0.8 V for E_u_∼82 meV and a 500 nm thick absorber. Correspondingly, the short‐circuit current for the case of a single junction cell (with back reflector) and top cell (no back reflector) are ∼24 mA/cm^2^ and ∼20 mA/cm^2^ as calculated from integrating absorptance and the AM1.5 solar spectrum. For a single junction cell, assuming fill factor of 80% would modestly achieve 15% efficiency. Moreover, the calculated top cell current density corresponds to the current density in the silicon bottom cell of a tandem. Considering the upper bound of 0.75 V for such a silicon bottom cell, the presented theoretical values for the Li_1.66_CuSn_3.33_S_8_ thin film already demonstrate the potential for increased efficiency by adopting a tandem structure with this material. Further technology optimization narrowing the E_u_ to 50 meV or 25 meV, would increase the maximum attainable V_OC_ to V_OC,EQE_∼1 and ∼1.2 V, respectively (see Supporting Information S1: Figure S6). Deep defects, by contrast, contribute slightly to the sub‐gap absorptance leaving V_OC_ unchanged, but can drastically reduce V_OC,EQE_ through the non‐radiative channel. Consequently, achieving suboptimal steepness of the absorption edge and reducing sulphur deficiency, which bears the defect chemistry of the absorber, defines the clear and primary direction for the future optimisation.

## 3 Conclusion

We report the first successful deposition of a nanocrystalline Lithium thiostannate thin film using the pulsed laser deposition technique, followed by post‐annealing at 400°C for 60 min. The key finding is that the phase purity of the PLD ablation target is a decisive factor in achieving the film crystallinity, morphology and optoelectronic properties. The phase‐pure film exhibits stoichiometry consistent with the target, with well‐resolved XRD reflections indexing to the *Fd* $\bar{3}$ *m* structure, a cubic parameter of *a* = 10.4(3) Å, consistent with the bulk phase and a pinhole‐free morphology (∼350 nm thickness). The respective spinel phase is relatively stable in ambient air and remains largely intact in DI water and dilute acid simultaneously. The optical absorption as well as photoconductivity spectra prove high absorption, similar to hybrid perovskite materials, with a bandgap of ∼1.5 eV, which is in excellent agreement with the photoluminescence at 836 nm. Measured photoconductivity and photoluminescence confirm non‐negligible carrier lifetime and mobility, proving the material's photosensitive character. The discussed limitations, including the sulphur deficiency and suboptimal steepness of the absorption edge, define the primary directions for future optimisation. Furthermore, being a member of the Li‐ion family, the potential of Li_1.66_CuSn_3.33_S_8_ for photo‐rechargeable batteries is tempting, but needs further investigation, for example careful evaluation of ionic conductivity. Overall, this work establishes Li_1.66_CuSn_3.33_S_8_ as a promising, non‐toxic and moisture‐stable photo‐absorber candidate for tandem photovoltaic applications.

## 4 Experimental Section

### 4.1 Thin Film Preparation

Reagents and synthesis: lithium sulphide (Li_2_S) (99.98% trace metals basis, Sigma‐Aldrich), copper sulphide (Cu_2_S) (99.99% trace metals basis, Sigma‐Aldrich), Tin sulphide (SnS) (99.5%, Lach‐Ner, Thermo‐Fisher) and sulphur (S) (99.999%, VWR) powders were used without further purification. The Li_1.66_CuSn_3.33_S_8_ precursor powder was mixed from Li_2_S, Cu_2_S, SnS and S powders in a 0.83: 0.5: 3.33: 6.67 M ratio in a nitrogen glovebox. The precursor powder mixture was then ground by a high‐energy ball mill for different durations to assess the evolution of homogeneity, density and porosity of the resulting powder. The powder milled for 3 hours and the phase‐pure thiospinel powder milled for 20 hours were pressed into cylindrical pellets using a stainless‐steel die with an inner diameter of 5 mm. A load of 0.8 tons was applied to produce pellets approximately 1–3 mm in height.

PLD film deposition: To prevent air exposure of the precursor materials (as the unreacted pellet was sensitive to air) and to minimise contamination of the PLD chamber, a compact portable vacuum chamber designed to fit inside the glovebox and compatible with the PLD setup was employed [33]. The pellet was placed on a holder plate at the bottom of this chamber, with substrates (quartz) positioned 55 mm above the pellet on a separate holder. After loading inside the glovebox, the chamber was transferred to the PLD system. It is connected to an external vacuum pump, enabling the deposition process in the chamber to be evacuated below 10^−4^ mbar. The PLD setup, manufactured by TSST B.V., is equipped with a KrF excimer laser (*λ* = 248 nm, COMPex 50) with a maximum pulse energy of 150 mJ and a pulse duration of 20 ns. Both the homogenised and phase‐pure Li_x_CuSn_3.33_S_z_ targets, as discussed above, were used for deposition and ablated using a KrF excimer laser with repetition rates ranging from 5 Hz, fluence 1.55 J cm^−2^ and laser energy set to 125 mJ. Each sample received 20,000 pulses, resulting in 300–400 nm thicknesses. Substrate heating during deposition was applied using a heater mounted above the substrate holder. Phase‐pure films were deposited with the substrate at 300°C, followed by in situ post‐annealing.

Post‐annealing: The deposited layer is further annealed in an inert atmosphere at higher temperatures (400°C). The annealing sequence consists of ramping up to the target temperature, then a period of constant temperature for 60 min and spontaneous cooling to room temperature after switching off the heating system. All films discussed in the main text were annealed at 400°C for 60 min.

### 4.2 Characterisations

*X‐ray diffraction (XRD):* The crystallinity of the thin films and pellets was characterised using a Rigaku SmartLab diffractometer with a rotating Cu anode and a nickel beta filter, operated in Bragg–Brentano geometry with adjustable slit settings. Each scan was performed over a period of 20 minutes, and repeated measurements confirmed the stability and reproducibility of the samples.

*RAMAN/PL spectroscopy:* The character and spatial distribution of individual chemical and crystalline phases in the sample were investigated using scanning Raman spectroscopy. A confocal Raman microscope (WITec alpha300 RAS) equipped with a green light laser (excitation wavelength 532 nm, laser power 1.0 mW). Raman spectra were collected by employing a 100x objective (NA 0.9) and a UHTS 300 VIS spectrometer. The typical time of spectra integration was 2 min. Spectra were collected and processed by WITec Control 6.1 and WITec Project 6.1 software. Lateral sample scanning was realised by an X‐Y piezo flexure stage, resulting in the Raman spectral maps in the areas of 20 × 20 μm^2^ with a scan speed of 60 s/line and a resolution of 150 × 150 pixels per image.

*X‐ray photoelectron spectroscopy (XPS):* The samples were mounted onto a sample holder in the glove box and transferred from the glove box to the XPS spectrometer using a plastic box under an argon‐inert atmosphere. The box with samples was introduced into a glove bag attached to the flexi lock of the spectrometer, filled with nitrogen. Nitrogen was continuously flowed through the bag during sample loading into the spectrometer, thereby minimising exposure to ambient air. XPS measurements were performed using an AXIS Supra photoelectron spectrometer (Kratos Analytical) equipped with a monochromatic Al Kα X‐ray source (hν = 1486.6 eV, 150 W). All spectra were acquired over an analysed area of approximately 1 mm^2^ under ultra‐high vacuum (UHV) conditions. The spectrometer energy scale was calibrated against the Au 4f_7/2_ core level at a binding energy of 83.9 eV. Surface charging artefacts were minimised using an electron flood gun, and all spectra were subsequently energy‐calibrated by referencing the Cu 2p_3/2_–932.2 eV. High‐resolution spectra were acquired for the Li 1s, Cu 2p_3/2_, Sn 3d_5/2_, S 2p, C 1s and O 1s core‐level regions using a pass energy of 40 eV and an energy step of 0.1 eV. Atomic concentrations were determined from integrated peak areas following Shirley background subtraction, applying element‐specific relative sensitivity factors (RSFs) as implemented in the ESCApe software (version 1.6.1.1234, Kratos Analytical).

*Scanning electron microscopy (SEM):* The surface morphology of all layers was characterised by Ultrahigh Resolution SEM (MAIA3, TESCAN) at an electron beam energy of 10 keV and using an in‐lens detector. All samples were in their pristine state without any conductive coating.

*Energy‐dispersive X‐ray spectroscopy (EDX):* For the determination of the chemical composition, energy‐dispersive X‐ray spectroscopy (EDX) was performed using a Thermo Scientific Phenom ProX SEM equipped with a Bruker XFlash 430 silicon drift detector and Bruker Quantax 70 software. Measurements were conducted at a beam voltage of 15 kV on the same instrument for all films. Elemental composition was obtained by averaging data from multiple areas and points of analysis on each sample.

*Photothermal deflection spectroscopy (PDS):* Optical absorption was deduced from the PDS measurements performed by a home‐built setup based on a He–Ne laser, 300 W Xe lamp, 3‐grating Andor Kymera 328i monochromator, NI PXIe‐4464 data acquisition card and software lock‐in analysis (modulation at ∼13.8 Hz). Spectral resolution was ΔE/E≈0.02, where *E* was photon energy. As the thermally sensitive liquid, Flutec PP1 with a refractive index of 1.25 was used. Simultaneously, transmittance and reflectance were measured. The absorption coefficient was evaluated from the absorptance‐to‐transmittance ratio according to equations from ref. [28] with the refractive index taken as constant. For photoconductivity measurements, the same setup was employed, where the signal was taken from a Keithley 428 preamplifier set at 10^9^ V/A instead of the PDS position detector. A 10 V DC bias was supplied by a Keithley 6517 electrometer.

## Author Contributions

**Naini Jain:** conceptualization, investigation, formal analysis, visualization, data curation, methodology, writing – original draft. **Kristina Vlášková:** writing – review and editing, data curation, formal analysis. **Lucie Landová:** data curation. **Neda Neykova:** data curation. **Dmytro Vorontsov:** data curation. **Oleksandr Romanyuk:** data curation, formal analysis. **Lukáš Horák:** data curation. **Milan Dopita:** data curation. **David Bušek:** resources. **Amalraj Peter Amalathas:** formal analysis, data curation. **Jakub Holovský:** supervision, resources, project administration, formal analysis, writing – review and editing, funding acquisition, conceptualization.

## Funding

This work was supported by the Czech Science Foundation (Grant No. 23‐06285S), by the project “The Energy Conversion and Storage”, funded as (Project No. CZ.02.01.01/00/22_008/0004617) by Programme Johannes Amos Comenius, call Excellent Research and by the Czech Technical University in Prague, student (Grant Nos. SGS24/135/OHK3/3T/13 and SGS26/129/OHK3/2T/13).

## Supporting information


Supporting Information S1

## Data Availability

The data that support the findings of this study are available from the corresponding author upon reasonable request.
