# Supplementary material for: Lithium Thiostannate Spinel Thin Films as Potential Photovoltaic Absorbers
Source: Nanophotonics. 2026 Oct 23;15(20):e70330. doi: 10.1002/nap2.70330 (PMC13692705; doi:10.1002/nap2.70330)
Supplement: Supplementary file 1 [file NAP2-15-e70330-s001.docx]

Supporting Information

**Lithium Thiostannate Spinel Thin Films as Potential Photovoltaic Absorbers**

*Naini Jain^1*^, Kristina Vlášková^1^, Lucie Landová^1,2^, Neda Neykova^1,2^, Dmytro Vorontsov^2^, Oleksandr Romanyuk^2^, Lukáš Horák^3^, Milan Dopita^3^, David Bušek^1^, Amalraj Peter Amalathas^1,4^, Jakub Holovský^1,2*^*

^1^SOL-MAT Lab, Faculty of Electrical Engineering, Czech Technical University in Prague, Technická 2, 166 27 Prague, Czech Republic, EU

^2^Institute of Physics, Czech Academy of Sciences, Cukrovarnická 10, 162 00 Prague, Czech Republic, EU

^3^Faculty of Mathematics and Physics, Charles University, Ke Karlovu 5, 121 16 Prague, Czech Republic, EU

^4^Department of Physics, Faculty of Science, University of Jaffna, Jaffna 40000, Sri Lanka

*Corresponding Author:* [jainnain@fel.cvut.cz](mailto:jainnain@fel.cvut.cz), jakub.holovsky@fel.cvut.cz

**Structural Characterisation of the pellet:**

|   **a.** |   **b.** |
| --- | --- |

**Figure S1 a.** XRD patterns of the homogenised and phase-pure pellets; **b.** Raman spectra of the phase-pure spinel pellet.

**XPS Analysis:**

**Figure S2.** High-resolution XPS spectra of the Sn 3d_5/2_, S 2p, O 1s, Cu 2p_3/2_, C 1s core levels and Cu LMM Auger spectra for the pressed powder sample (pellet, blue) and phase-pure Li_1.66_CuSn_3.33_S_8_ spinel Films (PLD film, purple). Similar line shapes and peak positions confirm similar atomic and electronic structures of the target and deposited film.

High-resolution XPS spectra reveal closely matching line shapes and peak positions for the phase-pure spinel pellet and the PLD-grown film. The strong spectral similarity across all core-level regions collectively confirms that the nominal chemistry of the target is preserved during the PLD process, with no evidence of significant compositional modification, secondary phase formation, or preferential elemental loss. A minor shift of the C 1s peak, within the range of 284.8-285.1 eV, is observed and is attributed to differential charging effects rather than changes in the carbon chemical environment.

The estimated surface contamination consists of approximately 45 at. % carbon and 10 at. % oxygen. Considering that oxygen associated with adventitious carbon typically accounts for ~25% of the carbon signal, it can be inferred that the majority of the detected oxygen is bonded to carbon-containing surface species. Nevertheless, lesser amounts of oxide species are present, as evidenced by the O 1s spectra. No sulphur oxide species were detected in the S 2p spectra. The dominant oxidation state of tin is Sn^4+^, while copper is present predominantly in the Cu(I) state (Cu^+^), as further confirmed by the position of the Cu Auger features. In addition, minor contributions from lithium oxide-related species were identified from the combined analysis of the Li 1s and O 1s spectral regions.

Taken together, the XPS results demonstrate that the PLD-deposited Li–Cu–Sn–S thin film faithfully reproduces the composition and electronic structure of the target pellet, validating PLD as an effective route for the preparation of phase-pure quaternary sulfide thin films. A surface contamination layer is present on both the pellet and film surfaces, however, it does not obscure the intrinsic chemical signatures of the Li–Cu–Sn–S phase.

**Optoelectronic Analysis:**

**Figure S3. a.** Tauc plot analysis; **b.** Urbach energy analysis using the absorption onset of phase-pure and non-phase-pure Li_1.66_CuSn_3.33_S_8_ films.

The reciprocity theorem, where the Urbach tail is not so steep, is as follows:

$\mathbf{PL}\left( \mathbf{E,}\boldsymbol{\triangle\mu} \right)\mathbf{=A}\boldsymbol{\propto}\frac{\boldsymbol{2}\boldsymbol{\pi A}\left( \boldsymbol{E,0} \right)\boldsymbol{E}^{\boldsymbol{2}}}{\boldsymbol{h}^{\boldsymbol{3}}\boldsymbol{c}^{\boldsymbol{2}}\left( \exp\left( \frac{\boldsymbol{E-\triangle\mu}}{\boldsymbol{KT}} \right)\boldsymbol{+1} \right)^{\boldsymbol{2}}}\boldsymbol{\propto}\frac{\boldsymbol{A(E,0)}\boldsymbol{E}^{\boldsymbol{2}}}{\left( \exp\left( \frac{\boldsymbol{E-\triangle\mu}}{\boldsymbol{KT}} \right)\boldsymbol{+1} \right)^{\boldsymbol{2}}}$	**(1)**

Where room-temperature blackbody radiation is calculated as 2𝜋ℎ𝑐^2^ / [𝜆^4^𝐸^2^(𝑒 ^𝐸⁄𝑘𝑇^ −1)], ℎ being the Planck constant, 𝑐 the speed of light, and 𝜆 the photon wavelength. This equation deals with macroscopic values of total absorptance and photoluminescence. Note that we have analysed the absorptance data to relate it to the emissivity of the photoluminescence, as shown in **Fig.S4.**

**Figure S4.** The green curve is the resulting curve from the calculation of the PL spectra using the reciprocity theorem, and the deep magenta dots are the PL spectra of the phase-pure Li_1.66_CuSn_3.33_S_8_ spinel films.

**Figure S5.** Photoluminescence spectra for non-phase and phase-pure Li_1.66_CuSn_3.33_S_8_ spinel films.

**Figure S6:** Simulated EQE (External Quantum Efficiency) for different cases of extrapolation of absorption edge. The curves were the input for the V_OC,EQE_ limit calculated according to ref ^[1]^ with assuming no localized defects and no light trapping.

**Stability Analysis:**

We performed the chemical stability test on our thin film in aqueous media of different pH values. Three Li_1.66_CuSn_3.33_S_8_ spinel films on quartz substrates were immersed in deionized (DI) water (Ph 7), HCl (0.1 M, Ph 1), and KOH (0.1 M, Ph 12) solution for 48 hours (see **Fig. S7**). The samples in the DI water and HCl solution showed no visible change over the course of the test (2 days). The film in KOH solution looked unchanged after 1 hr, but was visibly degraded after 24 hrs.

**Figure. S7.** Photographs of the Li_1.66_CuSn_3.33_S_8_ spinel film before and during the stability test (1, 24, and 48 hr) in DI water, HCl (0.1 M), and KOH (0.1 M).

Raman spectra were simultaneously performed after 1, 24, and 48 hrs immersion (see **Fig.S8 (a-c)**). The sample immersed in HCl solution showed the smallest spectral change: the main modes were retained even after 48 hr, with only a slight diminishing of the spectral band around 350 cm^-1^, which is commonly associated with Cu-S vibrations (**Fig. S8(a)**). This decrease may indicate a partial Cu-poor presence in the near-surface regions. In DI water, the intensity of the dominant mode (~318 cm^-1^), attributed mainly to Sn-S stretching, decreased(**Fig. S8(b)**). This is consistent with partial loss of Sn from the surface, or with surface oxidation or hydration of Sn-S bonds^[2]^. In KOH solution, all Raman modes weakened and broadened progressively at each measurement step (1, 24, and 48 hrs) (**Fig. S8(c)**). After 48 hr, no distinct spinel modes remained, indicating loss of long-range order together with visible film degradation.

**Figure. S8.** Raman spectra of the Li_1.66_CuSn_3.33_S_8_ spinel film before and during the stability test (1, 24, and 48 hrs) in; **a.** HCl (0.1 M), **b.** DI water, and **c.** KOH (0.1 M).

**References:**

[1]	J. Holovský, K. Ridzoňová, A. Peter Amalathas, B. Conrad, R. K. Sharma, X. Y. Chin, E. Bastola, K. Bhandari, R. J. Ellingson, S. De Wolf, *ACS Energy Lett.* **2023**, *8*, 3221.

[2]	Y. Morino, M. Otoyama, T. Okumura, K. Kuratani, N. Shibata, D. Ito, H. Sano, *ACS Omega* **2024**, *9*, 38523.
